# Erythropoiesis‐stimulating agents significantly delay the onset of a regular transfusion need in nontransfused patients with lower‐risk myelodysplastic syndrome

**DOI:** 10.1111/joim.12579

**Published:** 2016-12-07

**Authors:** H. K. G. Garelius, W. T. Johnston, A. G. Smith, S. Park, L. de Swart, P. Fenaux, A. Symeonidis, G. Sanz, J. Čermák, R. Stauder, L. Malcovati, M. Mittelman, A. A. van de Loosdrecht, C. J. van Marrewijk, D. Bowen, S. Crouch, T. J. M. de Witte, E. Hellström‐Lindberg

**Affiliations:** ^1^ Department of Medicine Section of Hematology and Coagulation Sahlgrenska University Hospital Gothenburg Sweden; ^2^ Epidemiology and Cancer Statistics Group Department of Health Sciences University of York York UK; ^3^ Clinique Universitaire d'hématologie CHU de Grenoble Université Grenoble Grenoble France; ^4^ Department of Hematology Radboud university medical center Nijmegen the Netherlands; ^5^ Service d'Hématologie Hôpital Saint‐Louis Assistance Publique des Hôpitaux de Paris (AP‐HP) and Université Paris 7 Paris France; ^6^ Department of Medicine Division of Hematology University of Patras Medical School Patras Greece; ^7^ Department of Hematology Hospital Universitario y Politécnico La Fe Valencia Spain; ^8^ Department of Clinical Hematology Institute of Hematology & Blood Transfusion Praha Czech Republic; ^9^ Department of Internal Medicine V (Hematology and Oncology) Innsbruck Medical University Innsbruck Austria; ^10^ Department of Hematology Oncology Fondazione IRCCS Policlinico San Matteo University of Pavia Pavia Italy; ^11^ Department of Medicine A Tel Aviv Sourasky (Ichilov) Medical Center Tel Aviv Israel; ^12^ Department of Hematology VU Institute of Cancer and Immunology VU University Medical Center Amsterdam the Netherlands; ^13^ St. James's Institute of Oncology Leeds Teaching Hospitals Leeds UK; ^14^ Department of Tumor Immunology Nijmegen Center for Molecular Life Sciences Radboud university medical center Nijmegen the Netherlands; ^15^ Department of Medicine Division of Hematology Karolinska Institutet Stockholm Sweden

**Keywords:** anaemia, haematology, haemoglobin, MDS, Myelodysplasia

## Abstract

**Background:**

The EUMDS registry is an unique prospective, longitudinal observational registry enrolling newly diagnosed patients with lower‐risk myelodysplastic syndrome (MDS) from 17 European countries from both university hospitals and smaller regional hospitals.

**Objective:**

The aim of this study was to describe the usage and clinical impact of erythropoiesis‐stimulating agents (ESAs) in 1696 patients enrolled between 2008 and 2014.

**Methods:**

The effects of ESAs on outcomes were assessed using proportional hazards models weighting observations by propensity to receive ESA treatment within a subset of anaemic patients with or without a regular transfusion need.

**Results:**

ESA treatment (median duration of 27.5 months, range 0–77 months) was administered to 773 patients (45.6%). Outcomes were assessed in 897 patients (484 ESA treated and 413 untreated). ESA treatment was associated with a nonsignificant survival benefit (HR 0.82, 95% CI: 0.65–1.04, *P* = 0.09); this benefit was larger amongst patients without prior transfusions (*P* = 0.07). Amongst 539 patients for whom response to ESA treatment could be defined, median time to first post‐ESA treatment transfusion was 6.1 months (IQR: 4.3–15.9 months) in those transfused before ESA treatment compared to 23.3 months (IQR: 7.0–47.8 months) in patients without prior transfusions (HR 2.4, 95% CI: 1.7–3.3, *P* < 0.0001). Responding patients had a better prognosis in terms of a lower risk of death (HR 0.65, 95% CI: 0.45–0.893, *P* = 0.018), whereas there was no significant effect on the risk of progression to acute myeloid leukaemia (HR 0.71, 95% CI: 0.39–1.29, *P* = 0.27).

**Conclusion:**

Appropriate use of ESAs can significantly delay the onset of a regular transfusion need in patients with lower‐risk MDS.

## Introduction

Lower‐risk myelodysplastic syndrome (MDS) is a malignant condition that is treated at both Medicine and Haematology Departments in small and large hospitals. The anaemia of patients with lower‐risk MDS has been associated with reduced quality of life in a number of small prospective Phase II trials 1, 2, 3, 4 and with reduced survival in retrospective registry reports 5. Current guidelines recommend erythropoiesis‐stimulating agents (ESAs) as first‐line treatment for patients with low‐ and intermediate‐1‐risk MDS with symptomatic anaemia 6, 7, 8, 9, 10; however, the effect on long‐term outcome in population‐based unselected cohorts is unknown. In recent studies, overall response rates varied between 38.0% and 65.5%, with a median response duration of around 20 months 2, 5, 11, 12. The efficacy of erythropoietin (EPO) can be enhanced by the addition of granulocyte colony‐stimulating factor (G‐CSF), mainly in MDS with ring sideroblasts; the median duration of response to the combined treatment is around 30 months, with some patients responding for more than 10 years 1. Patients with a low probability of response are characterized by a transfusion need exceeding 2 units per month combined with a serum EPO level of ≥500 U L^−1^ 3. However, some studies have shown that the effect of ESAs is decreased already at serum EPO levels of >100 U L^−1^ 5, 11, 13.

The French MDS (GFM) group compared overall survival in patients treated with ESAs within clinical studies with patients in the International Prognostic Scoring System (IPSS) database and showed a longer survival in the treated group 5. In another study, the same group reported that onset of a regular transfusion need was delayed if EPO treatment was started within 6 months of diagnosis 13. Another large retrospective study compared the outcome of patients treated within the Nordic EPO + G‐CSF studies 1990–1999 with that of untreated patients from the Italian Pavia registry recruited during the same period 2. The groups were matched for all major risk variables and very few patients received chelation treatment. Survival was markedly better in the group exposed to ESAs (RR 0.61, 95% CI: 0.44–0.83, *P* < 0.002) with no difference in transformation to acute myeloid leukaemia (AML). In a prospective clinical trial comparing EPO to supportive care in patients with lower‐risk MDS, the response rate was better in the EPO arm (36.0% vs. 9.6%) but the crossover design of the study prevented assessment of long‐term survival 14.

The European LeukaemiaNet MDS (EUMDS) registry is an unique, prospective, noninterventional longitudinal registry enrolling patients with lower‐risk MDS within 3 months of diagnosis from 17 countries across Europe 15. Therapy is given according to local guidelines. The main objective of this study was to explore the effects of ESAs on outcomes amongst patients with anaemia. The major and clinically highly relevant finding was that ESAs significantly prolong the time to first post‐ESA treatment transfusions in previously untransfused patients.

## Methods and materials

## Subjects

Patients newly diagnosed with IPSS low‐ or intermediate‐1‐risk primary MDS from university hospitals as well as from smaller regional units in a wide range of European countries were invited to participate as described elsewhere 15. Start and stop dates of treatment with ESAs or other MDS‐specific therapies, laboratory measurements, concomitant disease, medications and quality of life metrics were recorded approximately every 6 months until death or withdrawal from the study. Recruitment is ongoing with 1863 patients enrolled up to 16 April 2015. National ethics committees have approved the study, and all patients provided written informed consent for inclusion in the registry. At the start of the study, IPSS was the score system in use and only patients with IPSS low or intermediate‐1 scores were registered. The revised score (IPSS‐R) has since been established as the prefered scoring system and has been used in the present analyses including all registered patients despite some being identified by the IPSS‐R as not having low‐ or intermediate‐risk disease.

### ESA usage patterns

**Figure 1:**
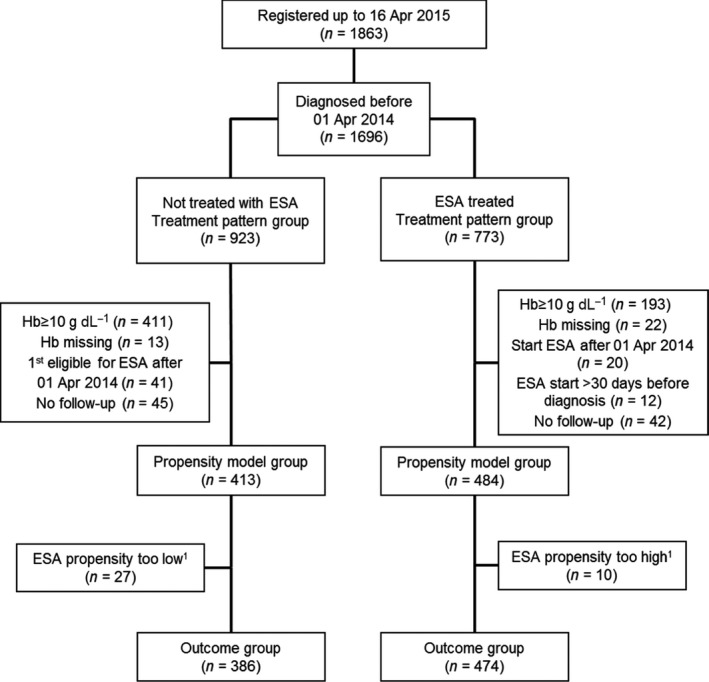
CONSORT diagram showing relationship between the groups of patients included in the different analyses presented in this study. The treatment pattern of erythropoiesis‐stimulating agents (ESAs) in different European countries was described for 1696 patients (773 ESA‐treated and 923 without ESA treatment). A total of 897 patients (484 ESA‐treated and 413 untreated) were retained for the *propensity model group*. The effect of ESA treatment on survival and disease progression was assessed only amongst the 860 analysed patients of the *outcome group* whose propensity scores for receiving ESA treatment were in the overlapping region of the propensity score distributions of treated and untreated patients. ^1^Patients with propensity scores outside the overlapping region denoted by the dashed lines in Fig. 4a. Hb, haemoglobin.

The patterns of ESA usage in the registry were described using univariable logistic regression. Only those patients diagnosed before 1 April 2014 (treatment usage group, *n* = 1696; Fig. 1) were included to avoid misclassification of ESA treatment.

### Response to ESA treatment

It was not possible to define response to ESAs by the International Working Group (IWG) 2006 criteria 16 as blood count data and transfusion information were recorded only every 6 months. Instead, response criteria were modelled on those of the Nordic 1, 17 and French 5 MDS groups. Briefly, patients were defined as responders if their haemoglobin (Hb) increased by at least 1.5 g dL^−1^ compared to the pre‐ESA treatment level. Additionally, patients who received transfusions prior to ESA treatment were defined as responders if no transfusions were administered between 8 and 16 weeks after receiving ESAs. Response rates were therefore underestimated using these compared to the IWG criteria (Supplementary material). Stopping ESA treatment within 8 weeks was interpreted as failure to respond. Importantly, remaining transfusion‐independent if ESA treatment was started prior to receiving any transfusions was not considered a response criterion. Response could be defined for 69.7% (*n* = 539) of all ESA‐treated patients. Direct adjustment of proportional hazards models for covariates (Supplementary material) was used to compare time‐to‐event outcomes of responding and nonresponding ESA‐treated patients.

### Effect of ESA treatment on patient outcomes

The effect of ESA treatment on survival and progression to AML was assessed amongst patients with a stable or a pretransfusion Hb value <10 g dL^−1^ at a visit before 1 April 2014 (*n* = 996). Patients starting ESAs >30 days prior to diagnosis with MDS (*n* = 12) and those only eligible for inclusion at their last recorded visit (*n* = 87) were excluded from this analysis, leaving 897 patients in the *propensity model group* (Fig. 1).

To overcome potential confounding by nonrandom allocation of ESA treatment, proportional hazards regression models comparing time‐to‐event outcomes in treated and untreated patients were weighted 18 by stabilized inverse probability of treatment weights 19 based on the propensity of a patient to receive ESA treatment. No further adjustments were included. Propensity to receive ESA treatment was modelled using multivariate logistic regression (Supplementary material). Only patients with comparable propensity scores were included in the analyses to estimate the effects of ESA treatment on outcomes (outcome group: *n* = 860; 474 treated and 386 not treated with ESAs; Fig. 1). The relationship between the effects of ESAs and pre‐ESA treatment transfusion status was explored with this model.

All analyses were performed using the SAS/STAT® software (SAS Institute Inc., Cary, NC, USA) 20, and tests of the proportional hazards assumption were undertaken in all analyses.

## Results

### Baseline characteristics of the cohort

The present analysis included 1696 patients diagnosed between January 2008 and April 2014 (Fig. 1). The median age at diagnosis was 74.4 years (range 18.7–95.3 years). The median follow‐up time amongst these patients, estimated by the reverse Kaplan–Meier method 21, was 3.7 years from diagnosis (range 0–7.0 years; 11 patients were included and censored on the date of diagnosis).

There was an overall male bias amongst the patients (61.0% men) in all World Health Organization (WHO) diagnostic groups with the exception of 5q‐syndrome (83.0% women). Amongst these diagnostic groups, refractory cytopenia with multilineage dysplasia (RCMD) was diagnosed most frequently (37.8%) followed by refractory anaemia (RA) (17.6%), RA with ring sideroblasts (RARS) (16.7%), RA with excess blasts (RAEB)‐1 (12.3%), 5q‐syndrome (6.1%), RCMD with ring sideroblasts (RCMD‐RS) (5.8%) and RAEB‐2 (0.4%). A further 3.2% (55 patients) had unclassified MDS (Table 1). The majority of patients were classified as low (42.1%) or very low (24.9%) risk according to the IPSS‐R (Table 1), with 15.0% classified as intermediate risk and 4.1% as high or very high risk and 13.0% were unclassified (<5% blasts and only one significant cytopenia).

**Table 1:** Baseline characteristics of patients diagnosed before 1 April 2014 showing distribution of factors by treatment with erythropoietin‐stimulating agents (ESA) and the association between each factor and the probability of receiving ESAs in a univariable logistic regression model

	Total (% of total)	ESA use		Odds ratio (95% confidence interval)	Likelihood ratio *P*‐value
		Treated (% of group)	Not treated (% of group)		
Total	1696 (100)	773 (45.6)	923 (54.4)		
Age at diagnosis (years)					
<60	190 (11.2)	62 (8.0)	128 (13.9)	0.48 (0.35, 0.67)	<0.0001
60–74	703 (41.5)	309 (40.0)	394 (42.7)	0.78 (0.64, 0.96)	
75+	803 (47.4)	402 (52.0)	401 (43.5)	1 (reference)
Sex					
Female	662 (39.0)	326 (42.2)	336 (36.4)	1.27 (1.05, 1.55)	0.015
Male	1034 (61.0)	447 (57.8)	587 (63.6)	1 (reference)	
MDS diagnosis (WHO 2008)					
RA	299 (17.6)	158 (20.4)	141 (15.3)	1.96 (1.49, 2.59)	<0.0001
RARS	283 (16.7)	163 (21.1)	120 (13.0)	2.38 (1.79, 3.16)
RCMD	641 (37.8)	233 (30.1)	408 (44.2)	1 (reference)	
RCMD‐RS	99 (5.8)	63 (8.2)	36 (3.9)	3.06 (1.97, 4.76)
RAEB‐1 or RAEB‐2	216 (12.7)	79 (10.2)	137 (14.8)	1.01 (0.73, 1.39)	
MDS‐U	55 (3.2)	21 (2.7)	34 (3.7)	1.08 (0.61, 1.91)
5q‐syndrome	103 (6.1)	56 (7.2)	47 (5.1)	2.09 (1.37, 3.17)	
IPSS‐R risk category					
Very low	423 (24.9)	155 (20.1)	268 (29.0)	0.48 (0.38, 0.62)	<0.0001
Low	714 (42.1)	389 (50.3)	325 (35.2)	1 (reference)
Intermediate	270 (15.9)	120 (15.5)	150 (16.3)	0.67 (0.5, 0.89)	
High/very high	69 (4.1)	30 (3.9)	39 (4.2)	0.64 (0.39, 1.06)
Unknown	220 (13.0)	79 (10.2)	141 (15.3)	0.47 (0.34, 0.64)	
Country					
Austria	95 (5.6)	31 (4.0)	64 (6.9)	0.42 (0.26, 0.67)	<0.0001
Croatia	3 (0.2)	0 (0.0)	3 (0.3)	–	
Czech Republic	124 (7.3)	39 (5.1)	85 (9.2)	0.39 (0.26, 0.6)	
Denmark	41 (2.4)	29 (3.8)	12 (1.3)	2.07 (1.03, 4.17)	
France	403 (23.8)	217 (28.1)	186 (20.2)	1 (reference)	
Germany	48 (2.8)	13 (1.7)	35 (3.8)	0.32 (0.16, 0.62)	
Greece	155 (9.1)	86 (11.1)	69 (7.5)	1.07 (0.74, 1.55)	
Israel	74 (4.4)	38 (4.9)	36 (3.9)	0.9 (0.55, 1.49)	
Italy	64 (3.8)	28 (3.6)	36 (3.9)	0.67 (0.39, 1.13)	
Netherlands	44 (2.6)	14 (1.8)	30 (3.3)	0.4 (0.21, 0.78)	
Poland	49 (2.9)	16 (2.1)	33 (3.6)	0.42 (0.22, 0.78)	
Portugal	35 (2.1)	17 (2.2)	18 (2.0)	0.81 (0.41, 1.62)	
Republic of Serbia	11 (0.7)	0 (0.0)	11 (1.2)	–	
Romania	40 (2.4)	24 (3.1)	16 (1.7)	1.29 (0.66, 2.49)	
Spain	131 (7.7)	71 (9.2)	60 (6.5)	1.01 (0.68, 1.51)	
Sweden	107 (6.3)	69 (8.9)	38 (4.1)	1.56 (1.00, 2.42)	
UK	272 (16.0)	81 (10.5)	191 (20.7)	0.36 (0.26, 0.5)	

MDS, myelodysplastic syndrome; ESA, erythropoiesis‐stimulating agent; WHO, World Health Organization; RA, refractory anaemia; RARS, refractory anaemia with ring sideroblasts; RCMD, refractory cytopenia with multilineage dysplasia; RCMD‐RS, refractory cytopenia with multilineage dysplasia and ringed sideroblasts; RAEB, refractory anaemia with excess blasts; MDS‐U, myelodysplastic syndrome, unclassifiable; IPSS‐R, revised International Prognostic Scoring System.

### Variation in the use of ESAs within Europe

In total, 773 patients (45.6% of all patients) were treated with ESAs, 57.8% of whom were male. Mean age in the treated and untreated groups were 71.7 and 74.3 years, respectively. Treated patients were older, and patients with higher MDS comorbidity index scores were more likely to receive ESAs than patients with low scores.

**Figure 2:**
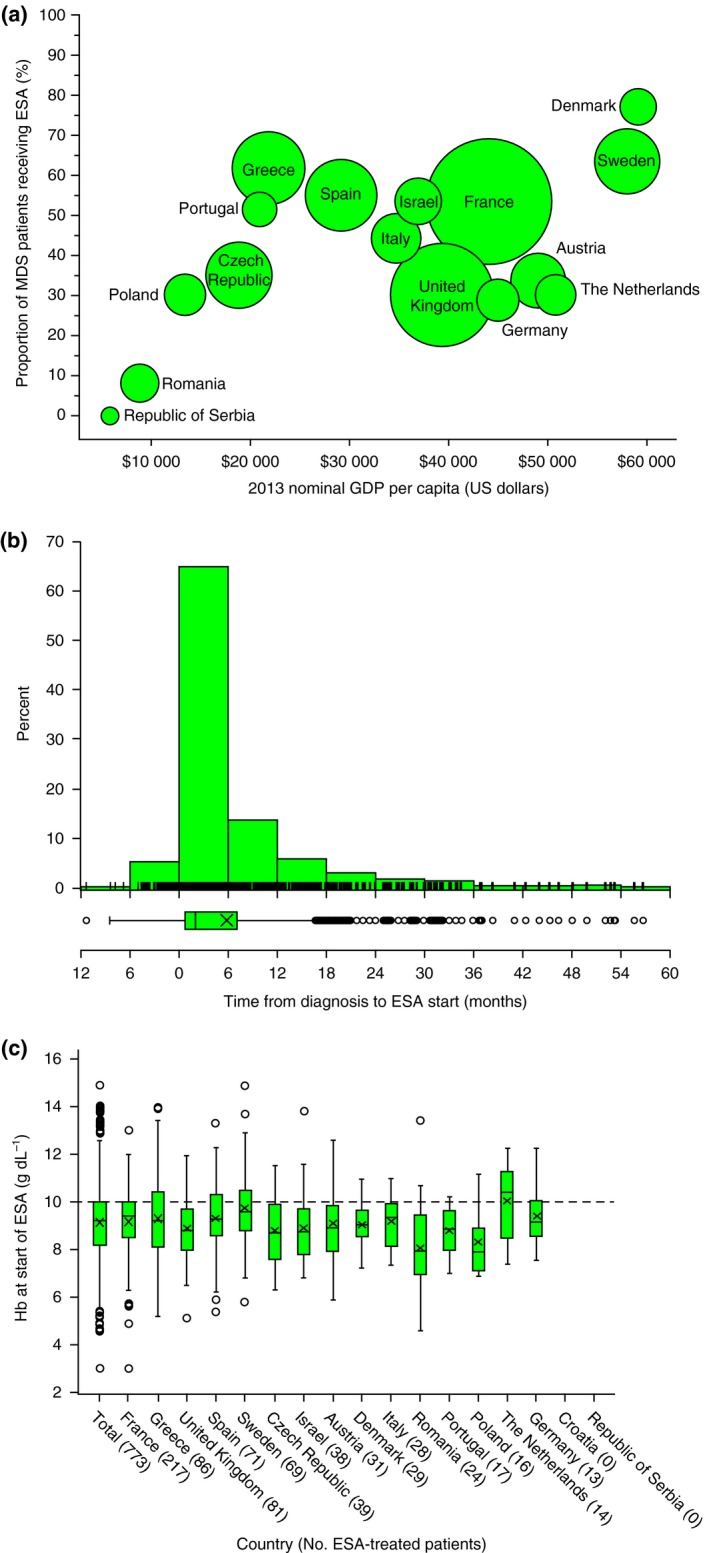
The use of erythropoiesis‐stimulating agents (ESAs) in a European low‐risk myelodysplastic syndrome (MDS) registry. (a) Proportion of registered patients receiving ESAs relative to the 2013 national gross domestic product (GDP) (source: http://www.imf.org/external/pubs/ft/weo/2014/02/weodata/index.aspx) showing no apparent relationship (bubble size is proportional to the number of registered patients shown in Table 1). (b) Most patients start ESA treatment relatively soon after diagnosis. (c) The haemoglobin (Hb) level at or near the start of treatment with ESAs amongst treated patients in participating countries was usually below the eligibility criteria of 10 g dL^−1^ (dashed line).

The proportion receiving ESAs varied by WHO classification and was highest amongst patients with RCMD‐RS (63.6%) and lowest amongst those with RCMD (36.3%; Table 1). ESA use was lowest in patients with a very low‐risk score (36.6%; Table 1). ESA use varied significantly between countries (Table 1), and there was no obvious relationship with national gross domestic product (GDP) (Fig. 2a) 22. Of all patients treated with EPO, 16.3% received parallel treatment with G‐CSF; patients with RCMD‐RS (27.0%) and RARS (20.9%) were the most likely to receive G‐CSF in addition to EPO, in line with previous reports 1, 2.

Amongst patients who started ESAs after diagnosis, the median time to treatment was 2.3 months (IQR: 0.9–7.6 months; Fig. 2b). The mean Hb value before the start of ESA treatment was 9.1 g dL^−1^ (range 3.0–14.9 g dL^−1^; Fig. 2c) with a substantial variation between countries. At the start of ESA treatment, 558 (72.2%) patients were defined as anaemic (Hb < 10 g dL^−1^ according to the WHO criteria); 287 of these patients received transfusions prior to starting ESAs. Of the remaining 215 ESA‐treated patients (27.8%), 160 patients started ESAs at a level of ≥10 g dL^−1^ Hb; the Hb level before the start of ESA treatment was not available in 22 patients and reflected previous transfusions in 33 patients. Once initiated, patients stayed on ESA treatment for a median of 27.5 months (range 0–77.0 months).

### Better long‐term outcome in patients with a response to ESA treatment

Using the criteria described in the Methods, a response to treatment could be assessed in 539 of the 773 ESA‐treated patients (69.7%) with 286 (53.1%) patients achieving a response, including 19 patients with ≥10 g dL^−1^ Hb at the start of ESA treatment and with an increase in Hb of ≥1.5 g dL^−1^. Responding patients had a better prognosis in terms of a lower risk of death (HR 0.65, 95% CI: 0.45–0.893, *P* = 0.018), whereas there was no significant effect on the risk of progression to AML (HR 0.71, 95% CI: 0.39–1.29, *P* = 0.27). The proportion of patients with an increase in Hb ≥1.5 g dL^−1^, or for whom blood transfusions were no longer required between 8 and 16 weeks after the start of ESA treatment varied depending on pre‐ESA treatment transfusion status: 26.7% and 74.2% amongst patients without and with pre‐ESA treatment transfusions, respectively. This is likely to reflect the fact that Hb levels were only recorded every 6 months, whereas all transfusions between visits were reported. When only a positive effect on Hb was taken as an indication of response, the response rate amongst those with no pre‐ESA treatment transfusions (27%) was comparable to the rate amongst those who received transfusions before ESA treatment (28%).

### Response to ESA treatment is associated with a delayed need for transfusion

**Figure 3:**
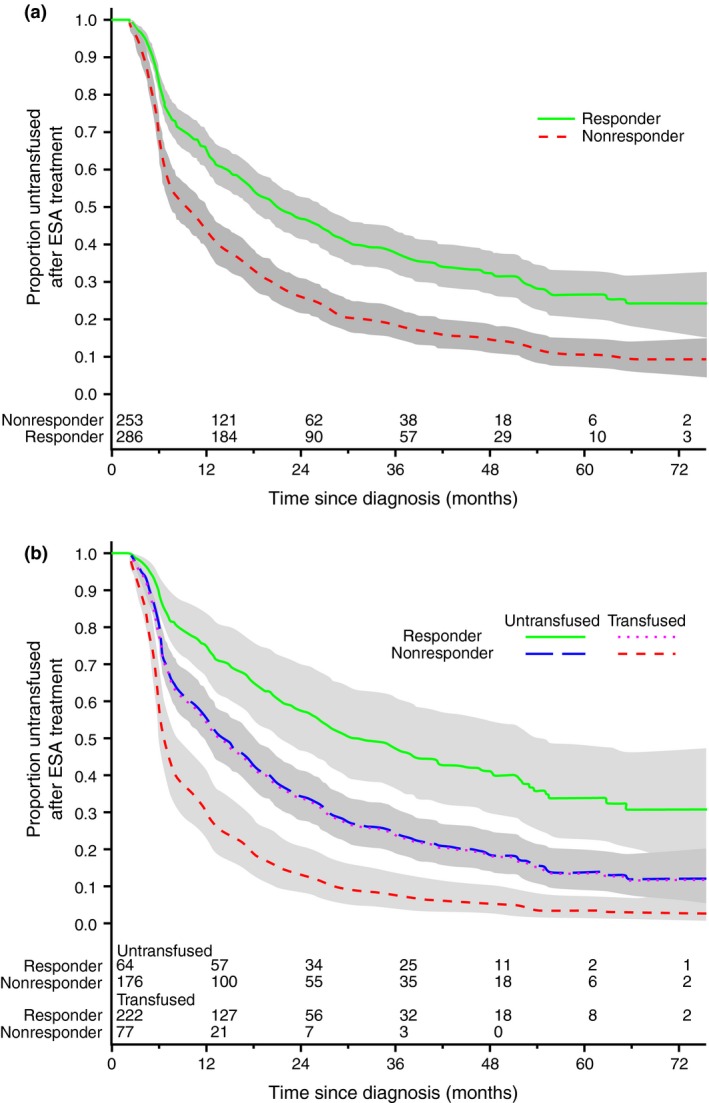
Comparison of time to first posterythropoiesis‐stimulating agent (ESA) treatment transfusion between ESA‐treated patients who did or did not respond to ESAs. (a) Time to first post‐ESA treatment transfusion was significantly improved amongst patients responding to ESA treatment compared to those not responding (HR 0.43, 95% CI: 0.32–0.57, *P* < 0.0001). (b) The response effect on time to first post‐ESA transfusion was evident when stratified by pre‐ESA transfusion experience (solid line versus long‐dashed line for untransfused patients and short‐dashed line versus dotted line for transfused patients).

When analysing patients irrespective of their pre‐ESA treatment transfusion status, time to the first post‐ESA treatment transfusion was longer amongst patients responding to ESAs (median transfusion‐free time 11.9 months) than amongst nonresponders (median 7.1 months; HR 0.43, 95% CI: 0.32–0.57, *P* < 0.0001; Fig. 3a). The effect of response on time to first post‐ESA treatment transfusion remained also after stratification by pretreatment transfusion experience (Fig. 3b). Importantly, and irrespective of response status, patients who received transfusions before starting ESAs had a shorter time to their first post‐treatment transfusion (median 6.1 vs. 23.3 months for nontransfused patients; HR 2.4, 95% CI: 1.75–3.31, *P* < 0.0001). Serum EPO measurements at or near the start of ESA treatment were available for 271 of the 539 patients (50.3%) with a defined response; median serum EPO amongst 153 responders (72.7 U L^−1^, IQR: 30–168 U L^−1^) was lower than amongst 118 nonresponders (100 U L^−1^, IQR: 38.4–218 U L^−1^); however, the difference was not significant (two‐sided Wilcoxon test, *P* = 0.113).

### Effect of ESA treatment on overall survival and risk of AML progression

**Figure 4:**
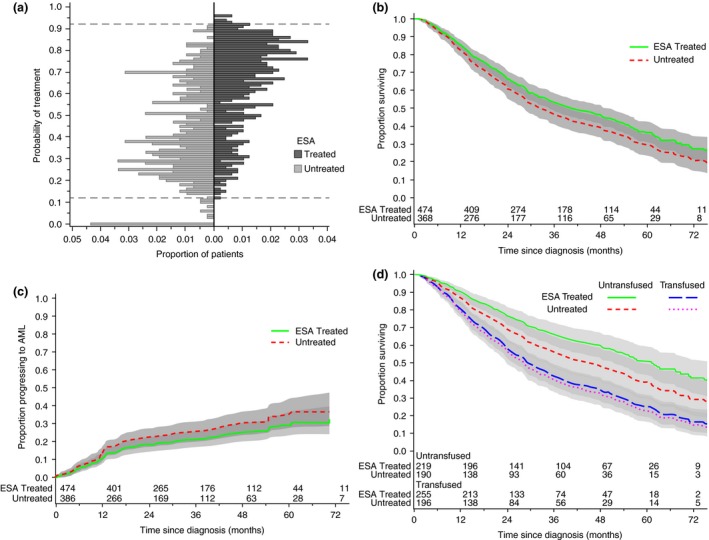
The effect of erythropoiesis‐stimulating agent (ESA) treatment on survival and progression to acute myeloid leukaemia (AML). (a) Distribution of propensity scores for ESA‐treated (dark grey bars) and untreated (light grey bars) patients showing upper and lower bounds of the overlapping region (dashed lines). (b) Estimated effect of ESA treatment on survival (HR 0.82, 95% CI: 0.65–1.03, *P* = 0.09). (c) Estimated effect of ESA treatment on progression to AML (HR 0.88, 95% CI: 0.63–1.22, *P* = 0.43). (d) estimated effect of ESA treatment on survival amongst patients not receiving transfusions before ESA treatment [treated (solid line) versus untreated (short‐dashed line): HR 0.71, 95% CI: 0.49–1.03, *P* = 0.070] was greater than amongst those who had at least one pre‐ESA treatment transfusion [treated (long‐dashed line) versus untreated (dotted line): HR 0.93, 95% CI: 0.70–1.26, *P* = 0.67]. There was no significant statistical interaction between these effects (*P* = 0.26).

In total, 897 patients (484 ESA treated and 413 untreated) were included in the *propensity model group* (Fig. 1). The strongest predictors of receiving ESAs were country and having a lower serum EPO level (Tables 2 and 3). The distributions of the propensity scores of ESA‐treated and untreated patients differed to a certain degree (Fig. 4a) but restricting the data set to treated and untreated patients with comparable propensity scores resulted in the loss of only 37 patients (Fig. 1) leaving 860 patients in the *outcome group* available for assessment of the effect of ESAs on disease progression and survival. Weighted comparisons of covariates in the propensity model (Tables 2 and 3) showed no differences between ESA‐treated and untreated patients.

**Table 2:** Outcome group: distribution of select categorical factors amongst 484 ESA‐treated and 413 untreated patients showing adjusted odds ratios and likelihood ratio test *P*‐values of the covariate effects from a multivariate logistic regression model of the propensity to receive ESA treatment

Level	Total (%)	Treated (%)	Untreated (%)	OR (95% CI)	Likelihood ratio test *P*‐value
Total	897 (100)	484 (100)	413 (100)	–	–
Sex					
Female	379 (42.3)	206 (42.6)	173 (41.9)	0.96 (0.69, 1.35)	0.834
Male	518 (57.7)	278 (57.4)	240 (58.1)	1 (reference)	
MDS diagnosis (WHO 2008)					
RA	135 (15.1)	87 (18.0)	48 (11.6)	0.53 (0.24, 1.17)	0.646
RCMD	336 (37.5)	154 (31.8)	182 (44.1)	0.41 (0.21, 0.83)
RARS	161 (17.9)	104 (21.5)	57 (13.8)	0.69 (0.33, 1.47)	
RCMD‐RS	58 (6.5)	40 (8.3)	18 (4.4)	1 (reference)
RAEB‐1 or ‐2	111 (12.4)	50 (10.3)	61 (14.8)	0.26 (0.09, 0.81)	
MDS‐U	26 (2.9)	11 (2.3)	15 (3.6)	0.32 (0.10, 0.96)
5q‐ syndrome	70 (7.8)	38 (7.9)	32 (7.7)	0.41 (0.17, 0.97)	
IPSS‐R category^a^					
Very low	129 (14.4)	60 (12.4)	69 (16.7)	1 (reference)	0.242
Low	429 (47.8)	261 (53.9)	168 (40.7)	1.69 (1.04, 2.74)
Intermediate	168 (18.7)	83 (17.1)	85 (20.6)	1.40 (0.76, 2.59)	
High/very high	57 (6.4)	24 (5.0)	33 (8.0)	1.21 (0.50, 2.90)
Unknown	114 (12.7)	56 (11.6)	58 (14.0)	1.23 (0.66, 2.28)	
Bone marrow blasts^a^					
<5%	781 (87.1)	428 (88.4)	353 (85.5)	1 (reference)	0.337
5–10%	116 (12.9)	56 (11.6)	60 (14.5)	1.59 (0.61, 4.14)
Serum erythropoietin^b^IU L^−1^					
≤32.4	87 (9.7)	52 (10.7)	35 (8.5)	1.64 (0.8, 3.36)	0.008
>32.4 to ≤64.0	91 (10.1)	53 (11.0)	38 (9.2)	1.42 (0.70, 2.91)	
>64.0 to ≤141.9	95 (10.6)	63 (13.0)	32 (7.7)	2.47 (1.23, 4.95)
>141.9 to ≤339.0	93 (10.4)	55 (11.4)	38 (9.2)	2.93 (1.44, 5.97)	
>339.0	93 (10.4)	41 (8.5)	52 (12.6)	1 (reference)
Missing	438 (48.8)	220 (45.5)	218 (52.8)	1.23 (0.70, 2.16)	
Transfusions prior to ESA treatment					
No	424 (47.3)	221 (45.7)	203 (49.2)	0.92 (0.64, 1.32)	0.656
Yes	473 (52.7)	263 (54.3)	210 (50.8)	1 (reference)	
MDS comorbidity index^b^					
Low	396 (44.1)	203 (41.9)	193 (46.7)	1 (reference)	0.705
Intermediate	328 (36.6)	190 (39.3)	138 (33.4)	1.06 (0.69, 1.62)	
High	173 (19.3)	91 (18.8)	82 (19.9)	0.88 (0.55, 1.40)	
Dyspnoea level^b^					
None	756 (84.3)	408 (84.3)	348 (84.3)	0.61 (0.27, 1.39)	0.221
Moderate	100 (11.1)	52 (10.7)	48 (11.6)	0.58 (0.23, 1.46)	
Slight	36 (4.0)	23 (4.8)	13 (3.1)	1 (reference)
Rest	5 (0.6)	1 (0.2)	4 (1.0)	0.08 (0.01, 0.94)	
Country					
Austria	45 (5.0)	17 (3.5)	28 (6.8)	1.40 (0.64, 3.07)	<0.001
Croatia	2 (0.2)	0 (0.0)	2 (0.5)	0 (–)
Czech Republic	86 (9.6)	29 (6.0)	57 (13.8)	0.66 (0.34, 1.29)	
Denmark	30 (3.3)	25 (5.2)	5 (1.2)	9.33 (3.13, 27.76)
France	167 (18.6)	123 (25.4)	44 (10.7)	5.56 (3.33, 9.28)	
Germany	28 (3.1)	9 (1.9)	19 (4.6)	0.78 (0.31, 1.99)
Greece	71 (7.9)	52 (10.7)	19 (4.6)	4.64 (2.23, 9.63)	
Israel	33 (3.7)	22 (4.5)	11 (2.7)	4.15 (1.65, 10.43)
Italy	25 (2.8)	15 (3.1)	10 (2.4)	2.88 (1.08, 7.67)	
Netherlands	21 (2.3)	6 (1.2)	15 (3.6)	0.70 (0.24, 2.05)
Poland	35 (3.9)	11 (2.3)	24 (5.8)	0.76 (0.32, 1.86)	
Portugal	24 (2.7)	15 (3.1)	9 (2.2)	2.41 (0.88, 6.61)
Romania	30 (3.3)	21 (4.3)	9 (2.2)	2.67 (0.96, 7.41)	
Republic of Serbia	8 (0.9)	0 (0.0)	8 (1.9)	0 (–)
Spain	61 (6.8)	38 (7.9)	23 (5.6)	2.79 (1.38, 5.64)	
Sweden	54 (6.0)	38 (7.9)	16 (3.9)	3.82 (1.78, 8.20)
UK	177 (19.7)	63 (13.0)	114 (27.6)	1 (reference)	

^a^At diagnosis; ^b^at the start of ESA treatment.

MDS, myelodysplastic syndrome; ESA, erythropoiesis‐stimulating agent; WHO, World Health Organization; RA, refractory anaemia; RARS, refractory anaemia with ring sideroblasts; RCMD, refractory cytopenia with multilineage dysplasia; RCMD‐RS, refractory cytopenia with multilineage dysplasia and ringed sideroblasts; RAEB, refractory anaemia with excess blasts; MDS‐U, myelodysplastic syndrome, unclassifiable; IPSS‐R, revised International Prognostic Scoring System.

**Table 3:** Distribution of noncategorical covariates amongst 484 ESA‐treated and 413 untreated patients, showing the likelihood ratio test *P*‐values for the covariates included in a logistic regression model of the propensity to receive ESA treatment

Covariate	ESA treatment	Mean (SD)	Minimum	Percentiles			Maximum	Likelihood ratio test *P*‐value
				25th	50th	75th		
Age (years)^a^^,^ b	Treated	73.7 (9.3)	41.4	67.9	74.4	80.6	95.3	0.48
	Untreated	72.3 (11.1)	21.0	64.9	74.2	80.5	94.0	
Time from diagnosis to ESA start (months)^b^	Treated	5.8 (9.0)	0.03	0.9	2.0	6.6	60.6	0.48
	Untreated	5.8 (9.8)	0	1.1	1.9	6.0	71.6	
Haemoglobin^b^^,^ c	Treated	8.5 (1.1)	4.6	7.9	8.7	9.4	10.0	0.29
	Untreated	8.5 (1.2)	0.6	7.8	8.7	9.4	10.0	
Cytopenias^c^^,^ d	Treated	1.5 (0.7)	1	1	1	2	3	0.25
	Untreated	1.6 (0.7)	1	1	1	2	3	
Karnofsky status^b^^,^ c ^,^ e	Treated	81.2 (13.7)	0	70	80	90	100	0.66
	Untreated	80.1 (15.5)	0	70	80	90	100	

^a^At diagnosis; ^b^fitted as b‐spline effect; ^c^at the start of ESA treatment; ^d^fitted as a linear effect; eimputed for 76 ESA‐treated and 46 untreated patients using a linear regression model including age at diagnosis, sex, country, the Sorror score and MDS comorbidity index and, if available, the dimensions and visual analogue score of the EQ‐5D questionnaire.

ESA, erythropoiesis‐stimulating agent; MDS, myelodysplastic syndrome.

A nonsignificant beneficial effect of ESA treatment on overall survival was estimated (HR 0.82, 95% CI: 0.65–1.04, *P* = 0.09; Fig. 4b) from the weighted regression model comparing patients with comparable propensity scores (Figs 1 and 4a). Progression to AML or high‐risk MDS was observed for 77 (16.2%) of the ESA‐treated patients and 66 (17.1%) of those not receiving ESAs; a nonsignificant estimate of a beneficial effect of ESA treatment was obtained (HR 0.88, 0.63–1.22, *P* = 0.44; Fig. 4c). The relationship between ESA treatment and pretreatment transfusion status was explored and revealed a larger estimated effect of ESAs on survival amongst patients who had not received transfusions prior to starting ESA treatment (treated compared to untreated: HR 0.71, 95% CI: 0.49–1.03, *P* = 0.07) than amongst patients who had received prior transfusions (treated compared to untreated: HR 0.93, 95% CI: 0.70–1.26, *P* = 0.67; Fig. 4d). The interaction between the effects did not add significantly to the regression model. Similar results were found when considering progression to AML (data not shown).

## Discussion

The aim of this study was to analyse treatment patterns of ESAs, as well as their effects on long‐term outcome in a large prospectively enrolled and well followed‐up cohort of patients with lower‐risk MDS. Because of the recruitment of patients from university hospitals as well as from smaller regional units in a wide range of European countries, our data can be viewed as representative of MDS patients in routine clinical practice. The higher median age of patients in the EUMDS registry (74.4 years) compared to other registries with a majority of patients from university hospitals (e.g. Düsseldorf, 72 years; Pavia, 65.3 years 23, 24) may be due to the wider recruitment. It may also be a reflection of an ageing population given the more recent establishment of the EUMDS registry.

Our results revealed marked variations in ESA use across Europe. Most but not all countries follow guidelines as recently proposed by the European LeukemiaNet 8. However, in some countries, transfusion need is a prerequisite for treatment initiation, an approach that is not supported by the findings of this analysis. Financial restrictions are placed on ESA use in certain countries including Poland, UK, the Netherlands and Greece, but overall, there was no apparent association between GDP and the use of ESAs, indicating that other factors also influence the therapeutic decision. Furthermore, there were marked variations in pre‐ESA treatment Hb levels between the countries, with Sweden and the Netherlands starting ESAs at higher Hb levels than for example Portugal, Poland and Romania, where patients were usually transfusion‐dependent before the start of treatment. It is interesting that across most countries, ESA treatment was more common amongst patients with, compared to those without, ring sideroblasts, reflecting a higher rate of symptomatic anaemia in this patient group but also more frequent use of other treatments, such as lenalidomide, in nonsideroblastic cases.

As Hb levels and number of transfusions were only assessed every 6 months, subtle changes in transfusion need and Hb levels used to identify haematological improvement as defined by IWG 2006 criteria 16 were not considered possible to assess. As a consequence of this, as response to ESAs is usually achieved within 8–12 weeks of starting treatment, subjects in the registry whose response had a duration of less than 6 months may have been misclassified as nonresponders using these criteria. Importantly, using these strict criteria, response rates are predicted to be lower than in prospective clinical trials. With this in mind, it is important to note that as high a proportion as 28% of transfusion‐dependent patients achieved both transfusion independency and a clear increase in Hb levels in response to treatment. The median treatment duration of 27.5 months indicates response duration of around 2 years, in line with previous reports 2. The responders received ESAs for a median duration of 38.3 months (IQR: 12.0–63.6 months), whereas nonresponders were treated for a median duration of 15.0 months (IQR: 3.3–44.6 months).

In most clinical trials and therapeutic guidelines, serum EPO appears to be a predictor of response to ESA treatment 3. This was also the case amongst those patients with available serum EPO measurements in this study, with only a few patients having serum EPO levels above 200 U L^−1^.

An important conclusion of this large observational registry study is that the response rate to ESAs as well as the capacity of these agents to significantly delay the onset of a regular transfusion need is most pronounced in transfusion‐naïve patients, thus corroborating the findings from a small retrospective study by the French GFM group 13. It could also be assumed that disease is more severe in patients with an urgent transfusion need and thus these patients are less likely to respond to ESAs; however, because several countries require a transfusion need before starting ESA treatment, it is likely that these differences partly reflect the fact that transfusion‐naïve patients are more responsive. In exploratory analyses, patients with a transfusion requirement of less than 2 units per month showed a pattern closer to that of the untransfused rather than transfused patients, but the groups were too small to allow for robust statistical comparison (data not shown). Clearly it would be desirable to prove this relationship in a prospective randomized study, but such a study would be difficult to conduct. Hence, we propose that ESAs should be recommended as first‐line treatment in low‐risk MDS patients with symptomatic anaemia before starting regular transfusions.

The plausibility of a country‐specific analysis was investigated; however, only two countries had sufficient comparable treated and untreated patients and therefore this issue may be considered in the future as the registry matures.

Previous retrospective epidemiological studies on cohorts collected within academic trials, compassionate use cohorts and the IPSS database 2, 5, 11 have shown significant survival benefits for ESA‐treated compared with untreated patients. It is clear that such studies carry inherent problems regarding patient selection. The prospective noninterventional EUMDS registry has several advantages and disadvantages but as treatment with ESAs is based solely on the physician's choice, it is most likely to be as close to the real‐world setting as possible, whilst still retaining control over objective entry and exit variables. As the decision to start ESA treatment could be based both on a too favourable clinical status and a too poor likelihood of responding to ESAs, we chose to base the outcome analysis on the propensity to receive ESAs 18. Our results showed a nonsignificant positive association between ESA treatment and overall survival (*P* = 0.09), but the difference between ESA‐treated and untreated patients was smaller than that observed in the above‐cited large retrospective studies.

Our data show that ESA treatment significantly delays the onset of a permanent transfusion need in all treated patient groups and in particular if initiated early. This is an important observation as a response to treatment with ESAs has been associated with improved quality of life in several studies 4, 12, 25. Moreover, blood transfusion due to bone marrow failure consumes valuable resources that could be allocated to other needs 26.

Despite treatment recommendation in most care programmes 6, 7, 8, this study shows that less than half of the MDS population receives ESAs at any time‐point. This seems to be due both to national financial and legal restrictions and to treatment traditions that do not follow European guidelines. However, with the marketing of several ESA biosimilars, the cost‐effectiveness of ESAs should improve with time, thereby shifting usage patterns towards the international recommendations.

Our findings demonstrate a marked variation in the usage pattern of recommended first‐line treatment for anaemic lower‐risk MDS. We conclude that this leads to clinically significant differences in the time to onset of a permanent transfusion need. Whether this has significant long‐term effects on overall survival, disease progression and time to initiation of other resource‐demanding treatments remains to be investigated. Clearly, the prospective design of the EUMDS registry provides information that could not be gained from conventional retrospective databases.

## Authors’ contributions

H.K.G.G., W.T.J., A.G.S. and E.H‐L. designed the study with input from A.A.vdL., S.P., L.dS., D.B., S.C., P.F and T.J.M.dW. H.K.G.G., W.T.J., A.G.S. and E.H.L. prepared the manuscript. W.T.J., together with A.G.S., performed all the statistical preparations. S.C. provided advice throughout the statistical process. The other authors are the principal investigators for their countries and have contributed patient material and data collection, and all authors contributed to critical revision of the manuscript; T.J.M.dW. is the principal investigator for the EUMDS study group.

## Conflict of interest statement

H.K.G.G. has received lecture honoraria from Celgene, Novartis and Alexion. A.G.S. has received research funding from Novartis, Cilag‐Janssen and Boehringer Ingelheim. S.P. has received research grants from Novartis, Celgene and Hospira. P.F. has received honoraria and research funding (as GFM chairman) from Celgene, Novartis and Amgen and is on advisory committees for Amgen, Böehringer‐Ingelheim, Celgene and Janssen. A.S. has received lecture honoraria, and consultancy reimbursement from Amgen, Celgene/GenesisPharma, Janssen‐Cilag, Gilead, Pfizer, MSD, Novartis and Genzyme/Sanofi. G.S. has received honoraria and research funding from Celgene, Novartis and Amgen and is on advisory committees for Amgen, Boehringer‐Ingelheim, Celgene, Merck Sharp and Dohme and Novartis. M.M has received research grants from Celgene (Neopharm), Johnson & Johnson, Novartis, Roche, GSK, Amgen (Medison). A. A.vdL. has received lecture honoraria from Celgene and Novartis and a research grant from Alexion. L.dS. and C.J.vM. are funded by the EUMDS project budget. T.J.M.dW. has received a honorarium from Novartis as the project coordinator of the EUMDS study, of which the present analysis is a part. E.H‐L. has received research funding from Celgene, however, not for this project. The remaining authors (J.C., R.S. and W.T.J., L.M., D.B. and S.C.) declare no competing financial interests.

## Financial support

The work of the EUMDS Registry for low and intermediate‐1 MDS has been supported by an unrestricted educational grant from Novartis Pharmacy B.V. Europe. This work is part of the MDS‐RIGHT activities, which has received funding from the European Union's Horizon 2020 research and innovation programme under grant agreement No 634789 ‐ “Providing the right care to the right patient with MyeloDysplastic Syndrome at the right time”. W.T.J., A.G.S. and S.C. are supported by Bloodwise (formerly Leukaemia & Lymphoma Research). Participants of the EUMDS registry receive financial support from the European Union's Horizon 2020 Research and Innovation Programme under grant agreement No 634789. ‘Providing the right care to the right patient with MyeloDysplastic Syndrome at the right time’ (MDS‐RIGHT).

## Supporting information


**Data S1.** Erythropoiesis stimulating agents significantly delay the onset of a regular transfusion need in previously non‐transfused patients with lower risk MDS and anemia. Supplementary information.
**Table S1** Days between the ‘before’ and ‘after’ ESA visit and the start of ESA treatment and months between these visits for patients with and without haemoglobin‐based response defined.
**Figure S1** Changes in Hb values before and after starting ESA among patients responding or not responding to ESA treatment. Vertical reference line denotes the start of ESA treatment and the horizontal reference line denotes Hb = 10 g dL^−1^. (a) Hb against time to/since starting ESA among Responders who had an increase in Hb of at least 1.5 g dL^−1^ between the visits. Blue lines indicate patients who changed from anaemic to non‐anaemic, green lines those who remained anaemic and red lines those who were initially non‐anaemic. (b) Hb against time to/since starting ESA among Non‐Responders.
**Figure S2** Time between start of ESA treatment the first post‐ESA transfusion for patients who had transfusions before starting ESA. Vertical reference lines indicate (from left to right) start of ESA, end of the first 8 weeks post‐ESA and the end of week 16 post‐ESA. Grey bars indicate patients who did not have a visit at least 16 weeks after starting ESA, blue bars indicate Non‐Responders, red bar indicate Responders.
**Table S2** Hemoglobin‐based response among all ESA‐treated patients.
**Table S3** Estimated follow‐up time in the study.
**Figure S3** Kaplan‐Meier estimates of ESA treatment duration among patients with Hb < 10 g dL^−1^ when they started ESA stratified by response status. Median duration among non‐responders = 14.4 months and among responders = 31.4 months.
**Table S4** Serum erythropoietin at the start of ESA or at the first 2 visits for patients not receiving ESA and transfusion experience up to the start of ESA or to the first 2 visit for patients not treated with ESA.
**Table S5** Use of other MDS‐specific treatments relative to treatment with ESA. Before = first use before ESA, With = evidence of overlap of the treatments, After = evidence of treatment the start of ESA.
**Figure S4** Receiver‐operator characteristic curve for the logistic regression model of the propensity to receive ESA treatment. A total of 484 treated and 413 untreated patients were included. Covariates as listed in Tables S1 and S2.
**Table S6** Results of χ^2^ and *t*‐tests comparing ESA treated patients to patients not treated with ESA with and without weighting by the propensity scores.
**Table S7** Summary hazard ratio estimates based on 25 imputed datasets carried through the analysis.
