# Supplementary material for: Erythropoiesis‐stimulating agents significantly delay the onset of a regular transfusion need in nontransfused patients with lower‐risk myelodysplastic syndrome
Source: J Intern Med. 2016 Dec 7;281(3):284–99. doi: 10.1111/joim.12579 (PMC5596334; doi:10.1111/joim.12579)
Supplement: Supplementary file 1 — Data S1. Erythropoiesis stimulating agents significantly delay the onset of a regular transfusion need in previously non‐transfused patients with lower risk MDS and anemia. Supplementary information. Table S1 Days between the ‘before’ and ‘after’ ESA visit and the start of ESA treatment and months between these visits for patients with and without haemoglobin‐based response defined. Figure S1 Changes in Hb values before and after starting ESA among patients responding or not responding to ESA treatment. Vertical reference line denotes the start of ESA treatment and the horizontal reference line denotes Hb = 10 g dL−1. (a) Hb against time to/since starting ESA among Responders who had an increase in Hb of at least 1.5 g dL−1 between the visits. Blue lines indicate patients who changed from anaemic to non‐anaemic, green lines those who remained anaemic and red lines those who were initially non‐anaemic. (b) Hb against time to/since starting ESA among Non‐Responders. Figure S2 Time between start of ESA treatment the first post‐ESA transfusion for patients who had transfusions before starting ESA. Vertical reference lines indicate (from left to right) start of ESA, end of the first 8 weeks post‐ESA and the end of week 16 post‐ESA. Grey bars indicate patients who did not have a visit at least 16 weeks after starting ESA, blue bars indicate Non‐Responders, red bar indicate Responders. Table S2 Hemoglobin‐based response among all ESA‐treated patients. Table S3 Estimated follow‐up time in the study. Figure S3 Kaplan‐Meier estimates of ESA treatment duration among patients with Hb < 10 g dL−1 when they started ESA stratified by response status. Median duration among non‐responders = 14.4 months and among responders = 31.4 months. Table S4 Serum erythropoietin at the start of ESA or at the first 2 visits for patients not receiving ESA and transfusion experience up to the start of ESA or to the first 2 visit for patients not treated with ESA. Table S5 Use of other MDS‐specific [file JOIM-281-284-s001.docx]

**Erythropoiesis stimulating agents significantly delay the onset of a regular transfusion need in non-transfused patients with lower-risk MDS**.

Hege KG Garelius, W. Thomas Johnston, Alexandra G Smith, Sophie Park, Louise de Swart, Pierre Fenaux, Argiris Symeonidis, Guillermo Sanz, Jaroslav Cermak, Reinhard Stauder, Luca Malcovati, Moshe Mittelman, Arjan A van de Loosdrecht, Corine van Marrewijk, David Bowen, Simon Crouch, Theo de Witte, and Eva Hellström-Lindberg for the EUMDS Group

**Supplementary information**

This supplementary information file is divided into eight sections. The first section defines response to ESA treatment as it could be determined in this dataset, the second examines the proportion of patients deemed to be responders and the third lists the covariates included in directly-adjusted proportional hazards regression models of data from only ESA-treated patients. Section 4 describes the amount of follow-up time available in this study. Section five comments on estimates of the duration of ESA treatment. The sixth section details serum erythropoietin and transfusion experience of patients receiving ESA when they started treatment and comparable data for patients not receiving ESA at each of their first two recorded visits. In section 7 is described other therapies received by patients in the study relative to when they started ESA treatment. The section provides additional detail of the propensity modelling exercise.

1. *Definition of response to ESA*

All patients, regardless of pre-ESA transfusion status, could be considered responders by their Hb increasing by at least 1.5 g/dL compared to their pre-ESA Hb regardless of Hb value when they started ESA (Figure S1A).

Patients with transfusions prior to receiving ESA could also be defined as responders if no transfusions were administered between 8 and 16 weeks after ESA treatment. However, remaining transfusion-independent if the patient had no pre-ESA transfusions was not considered a response criterion. Termination of ESA treatment within the first 8 weeks was interpreted as a failure to respond.

To define both a hemoglobin- and transfusion-based response a patient required two visits – one before and one after starting ESA.

As response to ESA is usually achieved within 8-12 weeks of starting treatment, responses lasting fewer than 6 months will be misclassified as non-response using these criteria. Consequently, the response rate may be lower compared to those reported in clinical trials. Furthermore, response status will be misclassified as undefined for non-responding patients who stop ESA before 8 weeks (for Hb-based response) or 16 weeks (for transfusion-based response) but who have not yet had a recorded visit after starting ESA.

The visits upon which the hemoglobin response was based had to occur within specific time windows with respect to the start of ESA treatment. The ‘before’ ESA measurement had to occur no more than 60 days before the start of ESA and no more than 7 days after the start of ESA. The ‘after’ ESA measurement had to occur at least 8 weeks after the start of ESA but no more than 1 year after the start of ESA. The distributions of the time to the visit before, visit after and the time between visits for all ESA treated patients for whom hemoglobin response could or could not be defined are given in Table S1.

| **Table S1:** Days between the ‘before’ and ‘after’ ESA visit and the start of ESA treatment and months between these visits for patients with and without haemoglobin-based response defined. | | | | | |
| --- | --- | --- | --- | --- | --- |
|  |  | Response Defined | | No Response defined | |
| Days to Visit before | n | 426 |  | 301 |  |
|  | mean (SD) | 25 | (29) | 93 | (60) |
|  | median (IQR) | 22 | (1, 37) | 91 | (64, 120) |
|  | missing | 3 |  | 43 |  |
|  |  |  |  |  |  |
| Days to Visit after | n | 427 |  | 291 |  |
|  | mean (SD) | 173 | (49) | 144 | (440) |
|  | median (IQR) | 168 | (147, 196) | 92 | (56, 130) |
|  | missing | 2 |  | 53 |  |
|  |  |  |  |  |  |
| Months between visits | n | 411 |  | 7 |  |
|  | mean (SD) | 6.49 | (1.39) | 5.53 | (1.68) |
|  | median (IQR) | 6.23 | (5.74, 7.05) | 5.77 | (3.77, 6.23) |
|  | missing | 2 |  | 353 |  |

| A | 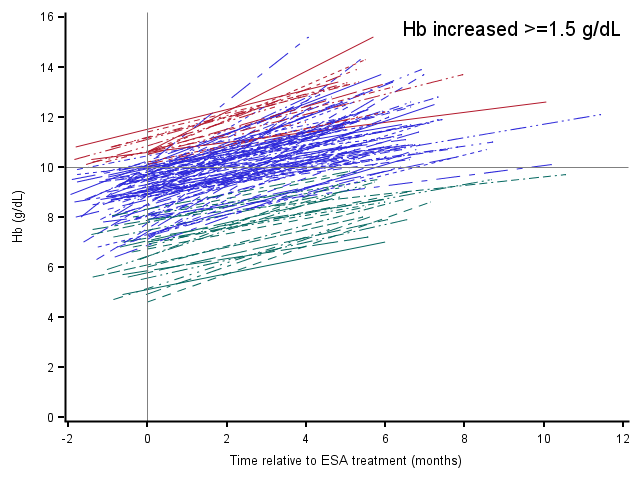 |
| --- | --- |
| B | 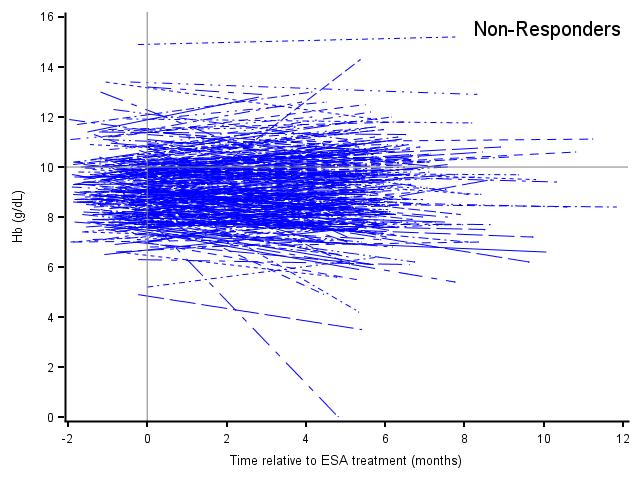 |
| **Figure S1: Changes in Hb values before and after starting ESA among patients responding or not responding to ESA treatment.** Vertical reference line denotes the start of ESA treatment and the horizontal reference line denotes Hb=10g/dL. A) Hb against time to/since starting ESA among Responders who had an increase in Hb of at least 1.5 g/dL between the visits. Blue lines indicate patients who changed from anaemic to non-anaemic, green lines those who remained anaemic and red lines those who were initially non-anaemic.B) Hb against time to/since starting ESA among Non-Responders. | |

For patients that had transfusions before starting ESA treatment another method was used to be defined as a Responder: If their need for transfusions was stopped for at least 8 weeks after the 8 weeks required for a response to ESA to develop, then they could be classified as a Responder. A patient had to have a visit at least 16 weeks follow-up after starting ESA (to account for the 8 weeks for the response to develop and another 8 weeks for the transfusion-free period) otherwise, transfusion-based response was undefined. In addition, all transfusions that occurred during the first 8 weeks after ESA treatment were ignored.

Transfusion information is gathered at each visit. For transfused patients the date of the last transfusion given is recorded; along with the total number of units received since the patient’s last visit. The first transfusion after the start date of ESA treatment plus 8 weeks was identified and if this transfusion occurred more than 16 weeks after the start of ESA treatment then the patient was classified as a Responder, otherwise as a Non-Responder. If no record of a transfusion was found, as long as the patient had a recorded visit at least 16 weeks after the start of ESA then the patient was deemed to be a Responder. Stopping ESA within 16 weeks of starting ESA was assumed to be an indicator of non-response.

There were 212 Transfusion-based Responders, 155 of these had times to their first post-ESA transfusion greater than 16 weeks after ESA treatment (Figure S2) . The other 57 had no evidence of a post-ESA transfusion, but had a post-ESA visit. Among the 87 Non-Responders, 14 had no evidence of a transfusion after the start of ESA plus 8 weeks, but these patients all stopped ESA within 16 weeks of starting. Four patients had a post-ESA transfusion reported at a visit that was not at least 16 weeks after starting ESA and were classified as Non-Responders. Two more patients that did not have a visit after the start of ESA plus 8 weeks, stopped ESA within 16 weeks and were classed as Non-Responders: the start and stop of ESA were disclosed at the same visit.

| 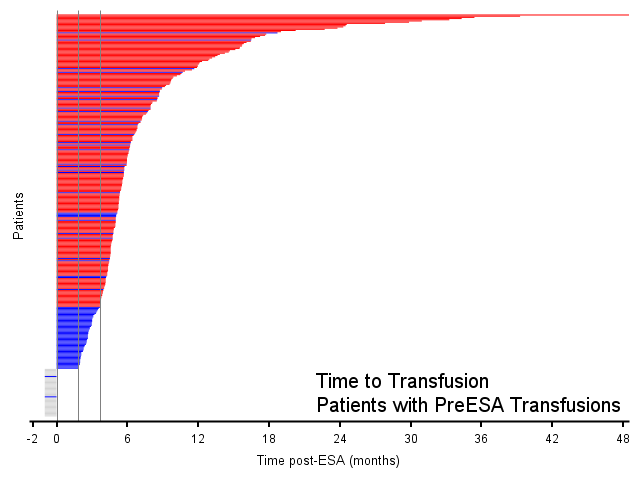 |
| --- |
| **Figure S2: Time between start of ESA treatment the first post-ESA transfusion for patients who had transfusions before starting ESA.** Vertical reference lines indicate (from left to right) start of ESA, end of the first 8 weeks post-ESA and the end of week 16 post-ESA. Grey bars indicate patients who did not have a visit at least 16 weeks after starting ESA, blue bars indicate Non-Responders, red bar indicate Responders. |

1. *Proportion defined as Responders*

Using the criteria defined above, response could be defined for 69.7% (539 patients) of the 773 ESA treated patients included in the present analysis. Among the 429 patients (55% of all ESA-treated patients) for whom hemoglobin-based response was defined, 117 (27%) were Responders (Table S2). This similar irrespective of of pre-ESA transfusion status: 27% among those patients without pre-ESA transfusions and 28% among those with pre-ESA transfusions. Hemoglobin-based response was slightly lower among patients with higher pre-ESA transfusion intensities (Table S2). The proportion of patients with a hemoglobin-based response decreased as serum EPO at the visit before starting ESA increased (Table S2).

There were 337 patients with pre-ESA transfusions and a transfusion-based response could be defined for 299 (89%) of them. Among these, 216 (72%) had a transfusion-based response. When split by transfusion intensity prior to ESA treatment, 128/174 patients (74%) with intensities <=2 units/month had a transfusion-based response whereas only 54/80 (68%) of patients with intensities >2 units/month were Responders.

When either definition of response was considered 286/539 (53%) of patients for whom response could be classified by either method were Responders.

| **Table S2:** Hemoglobin-based response among all ESA-treated patients | | | | |
| --- | --- | --- | --- | --- |
|  | | N | Responders | |
|  |  |  | n | % |
| Total | | 429 | 117 | 27 |
|  |  |  |  |  |
| Pre-ESA TFX experience | None | 240 | 64 | 27 |
|  | Any | 189 | 53 | 28 |
|  | ≤2 units/month | 97 | 28 | 29 |
|  | >2 units/month | 70 | 17 | 24 |
|  |  |  |  |  |
| Serum EPO | 3-28 | 43 | 20 | 47 |
|  | 28-56 | 40 | 13 | 33 |
|  | 57-106 | 42 | 15 | 36 |
|  | 107-229 | 51 | 10 | 20 |
|  | 231-2100 | 44 | 10 | 23 |
|  | Missing | 209 | 49 | 23 |

1. *Comparisons of outcomes in responders and non-responders*

Time-to-event comparisons between responding and non-responding patients utilized proportional hazards regression with direct adjustment for covariates. Factors included in the model included: country of registration, age at diagnosis, sex, MDS diagnosis, baseline revised IPSS-R category, bone marrow blast level at baseline, pre-ESA transfusion status, time between diagnosis and start of ESA, and the values of serum erythropoietin (categorized based on quintiles with a ‘missing’ category added), hemoglobin and number of cytopenias recorded when the patient started ESA.

1. *Distribution of length of follow-up*

All follow-up times were estimated using the reverse Kaplan-Meier method [1] which uses all events (deaths, progression to AML and withdrawal from the study) as censoring events for the observation time for the patient. The median follow-up time from diagnosis among the 1696 patients included in this analysis was 43.9 months (Table S3). Among the 897 patients in the propensity model group, the median follow-up time was 42.2 months and was marginally longer among ESA-treated patients than among those not receiving ESA (Table S3).

| **Table S3**: Estimated follow-up time in the study | | | | | |
| --- | --- | --- | --- | --- | --- |
|  | | All patients (n=1696)^1^ | *Propensity model group*^2^ | | |
|  |  |  | Total  (n=897) | ESA-treated (n=484) | Not ESA-treated (n=413) |
| Distribution (months) | Minimum | 0 | 0.03 | 0.03 | 0.4 |
|  | 25^th^ quantile | 22.1 | 23.2 | 25.2 | 20.4 |
|  | 50^th^ quantile | 43.9 | 42.2 | 44.3 | 39.9 |
|  | 75^th^ quantile | 58.6 | 57.1 | 59.1 | 56.2 |
|  | Maximum | 84.5 | 78.8 | 78.8 | 77.6 |
| Total person-years | | 3810 | 1779 | 1068 | 709 |
| ^1^ from date of diagnosis; ^2^ from ESA start date or first eligible visit for patients not treated with ESA | | | | | |

1. *Duration of ESA treatment*

Duration of ESA treatment was estimated to be 27.5 months (IQR: 8.6-63.6 months) among all patients who received ESA using the Kaplan-Meier method considering all trial outcomes as censoring events if stopping ESA treatment was not recorded in the registry. Among ESA-treated patients with Hb<10 g/dL when they started ESA, duration of treatment was a little shorter: median=25.9 months (IQR: 8.1-54.6 months). As might be expected, ESA treatment was shorter among patients not deemed to be responders (patients starting ESA with Hb<10 g/dL median=14.4 months (IQR: 3.5-42.7 months), Figure S3) than among patients with evidence of a response to ESA treatment (patients starting ESA with Hb<10 g/dL median=31.4 months (IQR: 10.4-63.6 months), Figure S3).

The estimate of duration of treatment among non-responders will be an overestimate related to our method of defining response. Firstly, our appraoch will result in patients with a short-term response being misclassified as non-responders as the average time between visits when measurements were compared was 6.49 months. This would be expected to increase the length of treatment among our non-responders by adding more patients with longer treatment durations. Secondly, a proportion true non-responders who have very short treatment times will be classed as undefined responders as they may not have had an appropriately-time follow-up visit yet. The impact of this would be to exclude a group of non-responders with the shortest treatment durations and thereby increase the median treatment duration of the remaining non-responders.

| 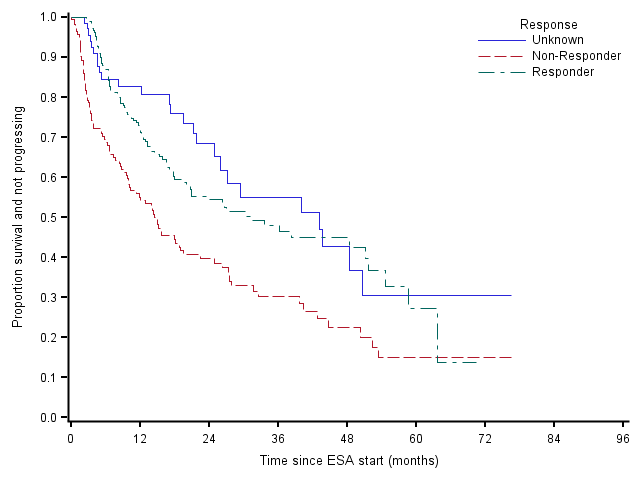 |
| --- |
| **Figure S3:** Kaplan-Meier estimates of ESA treatment duration among patients with Hb<10g/dL when they started ESA stratified by response status. Median duration among non-responders = 14.4 months and among responders = 31.4 months. |

1. *Serum Erythropoietin and transfusion experience*

| **Table S4:** Serum erythropoietin at the start of ESA or at the first 2 visits for patients not receiving ESA and transfusion experience up to the start of ESA or to the first 2 visit for patients not treated with ESA | | | | | | | | | | | | | | | | | | |
| --- | --- | --- | --- | --- | --- | --- | --- | --- | --- | --- | --- | --- | --- | --- | --- | --- | --- | --- |
|  | ESA-treated | | | | | | Not ESA treated | | | | | | | | | | | |
|  |  |  |  |  |  |  | At 1^st^ Visit | | | | | | At 2^nd^ Visit | | | | | |
|  | Total | | HB<10 | | hb>=10 | | Total | | HB<10 | | hb>=10 | | Total | | HB<10 | | hb>=10 | |
| n | 773 |  | 558 | (72.2) | 193 | (25.0) | 923 |  | 334 | (36.2) | 573 | (62.1) | 742 |  | 239 | (32.2) | 476 | (64.2) |
|  |  |  |  |  |  |  |  |  |  |  |  |  |  |  |  |  |  |  |
| Serum Erythropoietin (units) at ESA start or index visit | | | | | | | | | | | | | | | | | | |
| <=200 | 295 | (38.2) | 220 | (39.4) | 74 | (38.3) | 329 | (35.6) | 98 | (29.3) | 231 | (40.3) | 192 | (25.9) | 51 | (21.3) | 141 | (29.6) |
| >200 <=500 | 56 | (7.2) | 48 | (8.6) | 8 | (4.1) | 41 | (4.4) | 33 | (9.9) | 8 | (1.4) | 16 | (2.2) | 12 | (5.0) | 4 | (0.8) |
| >500 | 28 | (3.6) | 26 | (4.7) | 2 | (1.0) | 40 | (4.3) | 38 | (11.4) | 2 | (0.3) | 15 | (2.0) | 15 | (6.3) | 0 | (0.0) |
| missing | 394 | (51.0) | 264 | (47.3) | 109 | (56.5) | 513 | (55.6) | 165 | (49.4) | 332 | (57.9) | 519 | (69.9) | 161 | (67.4) | 331 | (69.5) |
|  |  |  |  |  |  |  |  |  |  |  |  |  |  |  |  |  |  |  |
| Transfusions prior to ESA or index visit | | | | | | | | | | | | | | | | | | |
| Untransfused | 436 | (56.4) | 257 | (46.1) | 160 | (82.9) | 704 | (76.3) | 130 | (38.9) | 558 | (97.4) | 519 | (69.9) | 66 | (27.6) | 429 | (90.1) |
| Any RBCT | 337 | (43.6) | 301 | (53.9) | 33 | (17.1) | 219 | (23.7) | 204 | (61.1) | 15 | (2.6) | 223 | (30.1) | 173 | (72.4) | 47 | (9.9) |
|  |  |  |  |  |  |  |  |  |  |  |  |  |  |  |  |  |  |  |
| Transfusion intensity (units/month) from 1^st^ transfusion to ESA start or index visit | | | | | | | | | | | | | | | | | | |
| <=2 | 191 | (24.7) | 181 | (32.4) | 10 | (5.2) | 124 | (13.4) | 116 | (34.7) | 8 | (1.4) | 151 | (20.4) | 108 | (45.2) | 40 | (8.4) |
| >2 | 89 | (11.5) | 84 | (15.1) | 5 | (2.6) | 77 | (8.3) | 72 | (21.6) | 5 | (0.9) | 66 | (8.9) | 61 | (25.5) | 5 | (1.1) |
| missing | 57 | (7.4) | 36 | (6.5) | 18 | (9.3) | 18 | (2.0) | 16 | (4.8) | 2 | (0.3) | 6 | (0.8) | 4 | (1.7) | 2 | (0.4) |

1. *Other treatments received*

| **Table S5:** Use of other MDS-specific treatments relative to treatment with ESA. Before=first use before ESA, With=evidence of overlap of the treatments, After=evidence of treatment the start of ESA. | | | | | | | | | | | | | | | |
| --- | --- | --- | --- | --- | --- | --- | --- | --- | --- | --- | --- | --- | --- | --- | --- |
| Treatment | ESA | All patients | | | | | | | Propensity model group | | | | | | |
|  |  | n | Before | | With | | After | | n | Before | | With | | After | |
| GCSF | Untreated | 923 | 5 | (0.5) | 15 | (1.6) | 16 | (1.7) | 413 | 2 | (0.5) | 12 | (2.9) | 12 | (2.9) |
|  | Treated | 773 | 14 | (1.8) | 126 | (16.3) | 145 | (18.8) | 484 | 8 | (1.7) | 82 | (16.9) | 106 | (21.9) |
| Hydroxyurea | Untreated | 923 | 3 | (0.3) | 5 | (0.5) | 5 | (0.5) | 413 | 3 | (0.7) | 5 | (1.2) | 5 | (1.2) |
|  | Treated | 773 | 6 | (0.8) | 13 | (1.7) | 22 | (2.8) | 484 | 4 | (0.8) | 5 | (1.0) | 21 | (4.3) |
| Demeth | Untreated | 923 | 7 | (0.8) | 22 | (2.4) | 22 | (2.4) | 413 | 4 | (1.0) | 14 | (3.4) | 14 | (3.4) |
|  | Treated | 773 | 5 | (0.6) | 26 | (3.4) | 54 | (7.0) | 484 | 2 | (0.4) | 14 | (2.9) | 39 | (8.1) |
| Immunosuppressive | Untreated | 923 | 3 | (0.3) | 18 | (2.0) | 18 | (2.0) | 413 | 3 | (0.7) | 17 | (4.1) | 17 | (4.1) |
|  | Treated | 773 | 4 | (0.5) | 16 | (2.1) | 19 | (2.5) | 484 | 3 | (0.6) | 11 | (2.3) | 15 | (3.1) |
| Prednisone | Untreated | 923 | 21 | (2.3) | 26 | (2.8) | 26 | (2.8) | 413 | 10 | (2.4) | 19 | (4.6) | 19 | (4.6) |
|  | Treated | 773 | 19 | (2.5) | 25 | (3.2) | 39 | (5.0) | 484 | 8 | (1.7) | 14 | (2.9) | 29 | (6.0) |
| New Agent | Untreated | 923 | 29 | (3.1) | 47 | (5.1) | 47 | (5.1) | 413 | 16 | (3.9) | 31 | (7.5) | 31 | (7.5) |
|  | Treated | 773 | 28 | (3.6) | 60 | (7.8) | 88 | (11.4) | 484 | 16 | (3.3) | 40 | (8.3) | 64 | (13.2) |
| Lenalidomide | Untreated | 923 | 4 | (0.4) | 25 | (2.7) | 25 | (2.7) | 413 | 2 | (0.5) | 22 | (5.3) | 22 | (5.3) |
|  | Treated | 773 | 9 | (1.2) | 33 | (4.3) | 58 | (7.5) | 484 | 4 | (0.8) | 21 | (4.3) | 52 | (10.7) |
| Any Chelator | Untreated | 923 | 9 | (1.0) | 48 | (5.2) | 49 | (5.3) | 413 | 7 | (1.7) | 45 | (10.9) | 46 | (11.1) |
|  | Treated | 773 | 9 | (1.2) | 66 | (8.5) | 115 | (14.9) | 484 | 8 | (1.7) | 50 | (10.3) | 94 | (19.4) |
| Transfusions | Untreated | 923 | 258 | (28.0) | 232 | (25.1) | 243 | (26.3) | 413 | 214 | (51.8) | 216 | (52.3) | 225 | (54.5) |
|  | Treated | 773 | 343 | (44.4) | 445 | (57.6) | 479 | (62.0) | 484 | 267 | (55.2) | 175 | (36.2) | 356 | (73.6) |
| Any Treatment | Untreated | 923 | 287 | (31.1) | 279 | (30.2) | 291 | (31.5) | 413 | 224 | (54.2) | 233 | (56.4) | 242 | (58.6) |
|  | Treated | 773 | 369 | (47.7) | 492 | (63.6) | 518 | (67.0) | 484 | 275 | (56.8) | 247 | (51.0) | 374 | (77.3) |

1. *Modeling the propensity to receive ESA treatment.*

To overcome potential confounding effects arising from non-random allocation of ESA treatment in this observational study proportional hazards regression models comparing time-to-event outcomes in treated and untreated patients were weighted [2] by stabilized inverse probability of treatment weights [3] based on the propensity to receive ESA with no further adjustments included in the outcome models. The propensity to receive ESA treatment among patients in the *propensity model group* (Figure 1; n=413+484=897) was modeled using multivariable logistic regression relating a range of factors suspected *a priori* to be involved in the decision to treat a patient with ESAs (Tables 2 and 3 of the main document) to ESA treatment status. Covariates in the model included both patient-related demographic factors and disease-related factors. All covariates listed in Table 2 were fit as categorical variables. The number of cytopenias and the Karnofsky status recorded at the start of ESA treatment (or first eligible visit for patients not receiving ESA – the term ‘start of ESA’ will be assumed to encompass this hereafter) were fitted as linear effects whereas age at diagnosis, the time between diagnosis and the start of ESA treatment and the hemoglobin recorded at the start of ESA treatment were all fitted as b-splines (Table 3).

Complete data were available for all covariates with the exception of Karnofsky status. For the 122 patients (76 ESA treated and 46 untreated) with missing Karnofsky status, it was initially predicted using a linear regression model that included age at diagnosis, sex, country, and the visual analog score, EQ-5D dimensions, Sorror co-morbidity score and MDS-comorbidity index at the start of ESA treatment. For those patients for whom the visual analog score and EQ-5D dimensions were not available, Karnofsky status was predicted using a linear regression model including only age at diagnosis, sex, country, and the Sorror score and MDS-comorbidity index. As a sensitivity analysis, a multiple imputation approach was also applied to these data and is summarized below.

Country of registration was one of the strongest predictors of receiving ESA treatment in the fitted logistic regression model (Table 2). In general, the probability of receiving ESA was higher among patients with lower serum erythropoietin levels (Table 2). No other factors had significant effects in the model (Tables 2 and 3). The model fitted the data adequately; the area under the ROC curve was 0.775 (Figure S4).

There was a qualitative difference in the distributions of the predicted propensity scores between the treated and untreated patients but there were treated patients with low probability of receiving treatment and untreated patients with high probability of receiving ESA (Figure 4A of main document). Restricting the dataset to patients with propensity scores in the common support region of the distribution (depicted by the horizontal lines in Figure 4A) resulted in the loss of 37 patients (10 ESA treated and 27 untreated patients).

Patient weights were derived from the predicted probability of receiving ESA treatment (the propensity score, π_i_) from the logistic regression model. To mitigate the influence of heavily weighted individuals, weights were stabilized using the marginal probability of treatment (p) [4]. For treated patients the weights (w_i_) were calculated as w_i_ = p / π_i_ and for untreated patients w_i_ = (1-p) / (1- π_i_). Covariate imbalance evident in unweighted comparisons of treated and untreated patients was eliminated when the comparisons were weighted (Table S6).

| 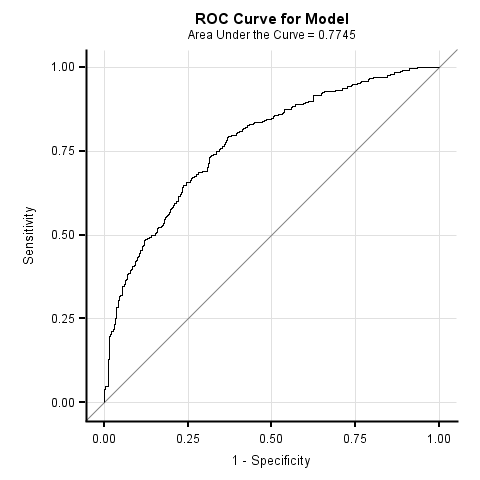 |
| --- |
| **Figure S4:** Receiver-operator characteristic curve for the logistic regression model of the propensity to receive ESA treatment. A total of 484 treated and 413 untreated patients were included. Covariates as listed in Tables S1 and S2. |

| **Table S6:** Results of χ^2^ and t-tests comparing ESA treated patients to patients not treated with ESA with and without weighting by the propensity scores. | | | | | | | | | | | | | |
| --- | --- | --- | --- | --- | --- | --- | --- | --- | --- | --- | --- | --- | --- |
| Factor type | Factor | Total dataset | | | | | | Truncated dataset^1^ | | | | | |
|  |  | Unweighted | | | Weighted | | | Unweighted | | | Weighted | | |
|  |  | Test Statistic | df | *p*-value | Test Statistic | df | *p*-value | Test Statistic | df | *p*-value | Test Statistic | df | *p*-value |
| Categorical^2^ | Country | 133.64 | 16 | <0.001 | 6.37 | 16 | 0.98 | 110.34 | 14 | <0.001 | 0.62 | 14 | 1 |
|  | Sex | 0.04 | 1 | 0.84 | 0.06 | 1 | 0.81 | 0.19 | 1 | 0.66 | 0.16 | 1 | 0.69 |
|  | WHO 2001 MDS Diagnosis | 32.47 | 6 | <0.001 | 0.98 | 6 | 0.99 | 26.96 | 6 | <0.001 | 0.62 | 6 | 1 |
|  | IPSS value^4^ | 16.75 | 4 | <0.001 | 0.28 | 4 | 0.99 | 13.4 | 4 | 0.01 | 0.46 | 4 | 0.98 |
|  | Bone marrow blasts^4^ | 1.73 | 1 | 0.19 | 0.12 | 1 | 0.73 | 1.6 | 1 | 0.21 | 0.16 | 1 | 0.69 |
|  | Ringed Sideroblasts^4^ | 16.23 | 1 | <0.001 | 0.54 | 1 | 0.46 | 12.15 | 1 | <0.001 | 0.17 | 1 | 0.68 |
|  | Serum Erythropoietin^5^ | 14.8 | 5 | 0.01 | 0.5 | 5 | 0.99 | 12.65 | 5 | 0.03 | 0.48 | 5 | 0.99 |
|  | Transfusion prior to ESA | 1.09 | 1 | 0.3 | 0.09 | 1 | 0.76 | 0.78 | 1 | 0.38 | 0.18 | 1 | 0.67 |
|  | MDS Comorbidity Index^5^ | 3.37 | 2 | 0.19 | 0.14 | 2 | 0.93 | 3.35 | 2 | 0.19 | 0.12 | 2 | 0.94 |
|  | Dyspnea level^5^ | 3.9 | 3 | 0.27 | 0.71 | 3 | 0.87 | 2.26 | 3 | 0.52 | 0.3 | 3 | 0.96 |
|  |  |  |  |  |  |  |  |  |  |  |  |  |  |
| Non-Categorical^3^ | Age at Diagnosis | -2.06 | 807.83 | 0.04 | -0.57 | 895 | 0.57 | -1.26 | 858 | 0.21 | -0.13 | 858 | 0.9 |
|  | Time Diagnosis to ESA start | 0.15 | 895 | 0.88 | 0.16 | 895 | 0.88 | -0.04 | 858 | 0.97 | 0.07 | 858 | 0.95 |
|  | Haemoglobin^5^ | -0.6 | 821.56 | 0.55 | -0.6 | 895 | 0.55 | -0.09 | 858 | 0.92 | -0.38 | 858 | 0.71 |
|  | Cytopenias^5^ | 3.59 | 836.44 | <0.001 | 0.34 | 895 | 0.73 | 3.25 | 858 | <0.001 | 0.12 | 858 | 0.9 |
|  | Karnofsky status^5^ | -1.05 | 828.94 | 0.29 | 0.03 | 895 | 0.98 | -0.76 | 858 | 0.45 | 0.19 | 858 | 0.85 |
| ^1^ only includes patients form the common support region of the distribution of propensity scores; ^2^ compared using χ^2^ test; ^3^ compared using a t-test; ^4^ At diagnosis; ^5^ At start of ESA treatment | | | | | | | | | | | | | |

Karnofsky status was derived using multiple imputation using chained equations[5] . Propensity modelling, calculation of weights and proportional hazards modelling were all carried out for each of 25 imputed datasets. The ESA effect estimates were then summarized across the 25 analyses (Table S7). The resulting estimates and uncertainty were comparable to that arising from the simpler analysis detailed in the main paper.

| **Table S7:** Summary hazard ratio estimates based on 25 imputed datasets carried through the analysis | | | | |
| --- | --- | --- | --- | --- |
| Outcome | Summary Hazard ratio (95% CI) | | p | Range of Hazard ratio estimates |
| Overall survival | 0.83 | (0.66, 1.04) | 0.106 | (0.81, 0.84) |
| Disease progression | 0.8 | (0.54, 1.18) | 0.259 | (0.79, 0.81) |

**References**

1 Schemper M, Smith T. A note on quantifying follow-up in studies of failure time. *Control Clin Trials* 1996; **Aug;17:** 343-6.

2 Austin P. The use of propensity score methods with survival or time-to-event outcomes: Reporting measures of effect similar to those used in randomized experiments. *Stat Med* 2014; **Mar 30:** 1242-58.

3 Cole SR, Hernan M. Adjusted survival curves with inverse probability weights. *Comput Methods Programs Biomed* 2004; **Jul;75.:** 45-9.

4 Robins JM, Hernan M, Brumback B. Marginal Structural Models and Causal Inference in Epidemiology. *Epidemiology* 2000; **11:** 550-60.

5 Inc. SSSI. *software 14.1 User's Guide TEC, NC:*. 2015.
